# Caco-2 Cell Conditions Enabling Studies of Drug Absorption from Digestible Lipid-Based Formulations

**DOI:** 10.1007/s11095-017-2327-8

**Published:** 2018-02-26

**Authors:** Janneke Keemink, Christel A. S. Bergström

**Affiliations:** Department of Pharmacy, Uppsala Biomedical Center, Uppsala University, P.O. Box 580, SE-751 23 Uppsala, Sweden

**Keywords:** Caco-2 cells, digestion, intestinal absorption, lipid-based formulation

## Abstract

**Purpose:**

To identify conditions allowing the use of cell-based models for studies of drug absorption during *in vitro* lipolysis of lipid-based formulations (LBFs).

**Methods:**

Caco-2 was selected as the cell-based model system. Monolayer integrity was evaluated by measuring mannitol permeability after incubating Caco-2 cells in the presence of components available during lipolysis. Pure excipients and formulations representing the lipid formulation classification system (LFCS) were evaluated before and after digestion. Porcine mucin was evaluated for its capacity to protect the cell monolayer.

**Results:**

Most undigested formulations were compatible with the cells (II-LC, IIIB-LC, and IV) although some needed mucin to protect against damaging effects (II-MC, IIIB-MC, I-LC, and IIIA-LC). The pancreatic extract commonly used in digestion studies was incompatible with the cells but the Caco-2 monolayers could withstand immobilized recombinant lipase. Upon digestion, long chain formulations caused more damage to Caco-2 cells than their undigested counterparts whereas medium chain formulations showed better tolerability after digestion.

**Conclusions:**

Most LBFs and components thereof (undigested and digested) are compatible with Caco-2 cells. Pancreatic enzyme is not tolerated by the cells but immobilized lipase can be used in combination with the cell monolayer. Mucin is beneficial for critical formulations and digestion products.

**Electronic supplementary material:**

The online version of this article (10.1007/s11095-017-2327-8) contains supplementary material, which is available to authorized users.

## Introduction

Drug dissolution in gastrointestinal fluids is crucial for drug absorption. However, approximately 70% of new drug candidates show insufficient solubility to allow intestinal absorption (1). Therefore, formulation strategies have been developed to improve bioavailability. Of these, lipid-based formulations (LBFs) often provide a means to deliver highly lipophilic, poorly water-soluble compounds at concentrations high enough to support absorption. These formulations consist of various mixtures of oils, surfactants, and co-solvents and are classified according to their composition and physical characteristics in the lipid formulation classification system (LFCS) (2). Ultimately, LBFs are employed to keep the compounds in solution during their transit in the gastrointestinal tract and expose the absorptive site to drugs in a solubilized and/or supersaturated state.

However, upon oral administration, many of the formulation components undergo lipolysis which changes the solvation capacity of the medium keeping the drugs in solution. To simulate and study this digestion process *in vivo*, an *in vitro* lipolysis model is commonly used (3). This model allows estimation of (i) the extent of digestion, and (ii) the drug distribution between the three phases present in the gastrointestinal tract (i.e. oil, aqueous and solid/precipitated drug, phases). Unfortunately, these studies do not quantitatively predict the *in vivo* performance of the drugs. Rather, they provide rank order correlations, and it has been speculated that this is due to the absence of an absorptive sink (3–6). During *in vitro* lipolysis experiments, the drug is not transported away from the solution as would be the case if it was absorbed. This artifact drives higher supersaturation levels leading to precipitation *in vitro* that would not occur *in vivo* (7).

Previous work has addressed this issue. For example, biopharmaceutical modeling has been used to predict intestinal absorption using *in vitro* lipolysis data (8). Permeation studies have also been performed on intestinal tissue of rats with predigested LBFs (9). Both methods seem promising but do not capture the dynamics of the *in vivo* processes. Recently, Crum *et al*. suggested a new animal-based model coupling *in situ* intestinal perfusion in rats to the *in vitro* digestion of LBFs (10). This method provides real time observations, but is time consuming and, since it is animal-based, is mainly suited for mechanistic studies rather than routine screening.

The use of Caco-2 cells in-line with lipolysis would offer an easier and faster approach than *in situ* animal studies. Caco-2 cells are a human colon carcinoma cell line considered the gold standard for the assessment of oral drug absorption nowadays (11). Differentiated Caco-2 cells resemble the epithelium of the human intestine and allow the prediction of drug transport mediated by different pathways, e.g., passive and active transport via the para- and transcellular routes (12). However, digestion media used in lipolysis experiments have been shown to damage Caco-2 cells (13–15). The aim of this study was therefore to evaluate compatibility between Caco-2 cells and individual components present during *in vitro* digestions to identify conditions under which Caco-2 cells can be used in a new *in vitro* model that simultaneously investigates digestion and absorption of compounds present in LBFs. A protective layer of mucin was used to increase biorelevance and generate Caco-2 compatibility.

## Materials and Methods

### Materials

All culture media and supplements were purchased from Invitrogen AB (Sweden). [^14^C]-mannitol was purchased from PerkinElmer Sverige AB (Sweden); Novozym® 435 (immobilized lipase) was obtained from Strem chemicals (France); and fasted state simulated intestinal fluid (FaSSIF) powder was obtained from biorelevant.com (UK). Tris-maleate, CaCl_2_.2H_2_O, NaCl, NaOH, oleic acid, caprylic acid, mucin from porcine stomach type III, Soybean oil, Cremophor EL, Tween 85, PEG400, Carbitol, and porcine pancreatin extract (8× USP specifications activity) were purchased from Sigma-Aldrich (USA). Maisine 35–1 was a kind gift from Gattefossé (France), and Captex 355 and Capmul MCM were kind gifts from Abitec (USA). Excipient details can be found in Table I.

**Table I:** Name and Composition of Excipients Used in the Investigated Lipid-Based Formulations

Excipients	Composition
Triglycerides	
Soybean oil	LC triglycerides: predominantly linoleic acid, linolenic acid, oleic acid, stearic acid and palmitic acid
Captex 355	MC triglycerides: predominantly glycerol tricaprylate (C8)/caprate (C10)
Mixed glycerides	
Maisine 35–1	LC glycerides: 33.5% monoglyceride, 50.9% diglyceride and 14.7% triglyceride; predominantly linoleic acid (C18) and oleic acid (C18)
Capmul MCM	MC glycerides: 60.7% monoglyceride, 33.1% diglyceride and 4.4% triglyceride predominantly caprylic acid (C8) and capric acid (C10)
Surfactants	
Cremophor EL	polyethoxylated castor oil (HLB 14–16)
Tween 85	polyoxyethylenesorbitan triolate (HLB 11)
Cosolvents	
PEG 400	polyethylene glycol 400
Carbitol	diethylene glycol ethyl ether

Abbreviations: *MC* Medium-chain, *LC* Long-chain

### Lipid-Based Formulations

Nine formulations were chosen to represent the four LFCS classes (16); these contained long-chain (LC) or medium-chain (MC) glycerides, surfactants, and co-solvents (Table II). The LBFs resemble formulations that were previously used to develop a standardized *in vitro* lipolysis method (17), and were herein selected to allow future comparisons between this standardized method and a potential digestion-absorption method to be developed based on the results obtained in the current study. Formulations were prepared as described previously (18). Briefly, excipients were pre-heated (37°C, except for Maisine 35–1 70°C) and weighed into glass vials according to predefined fractions (% *w*/w; Table II). Subsequently, vials were sealed, vortex mixed and placed on a shaker (300 rpm), at 37°C for 24 h.

**Table II:** Composition of Investigated LBFs, Representing All Classes of the Lipid Formulations Classification System

Formulation	Composition (% *W*/W)	
I-MC	50%	Captex 355
	50%	Capmul MCM
II-MC	32.5%	Captex 355
	32.5%	Capmul MCM
	35%	Tween 85
IIIA-MC	32.5%	Captex 355
	32.5%	Capmul MCM
	35%	Cremophor EL
IIIB-MC	12.5%	Capmul MCM
	12.5%	Captex 355
	25%	Carbitol
	50%	Cremophor EL
I-LC	50%	Soybean oil
	50%	Maisine 35–1
II-LC	32.5%	Soybean oil
	32.5%	Maisine 35–1
	35%	Tween 85
IIIA-LC	32.5%	Soybean oil
	32.5%	Maisine 35–1
	35%	Cremophor EL
IIIB-LC	5%	Soybean oil
	45%	Tween 85
	50%	Carbitol
IV	50%	Cremophor EL
	50%	Carbitol

I–IV denotes the type of lipid based formulation according to the lipid formulation classification system

Abbreviations: *MC* Medium-chain, *LC* Long-chain

### Preparation of Pancreatic Extract

Pancreatic extract was prepared by mixing 0.6 g pancreatin powder with 3 mL of lipolysis buffer containing 2 mM Tris-maleate, 1.4 mM CaCl_2_, and 150 mM NaCl (pH 6.5) followed by centrifugation for 15 min at 21,000 *g* and 5°C (17). Subsequently, the extract was diluted with digestion medium (lipolysis buffer supplemented with FaSSIF powder to obtain sodium taurocholate concentrations of 3.0 mM and lecithin concentrations of 0.75 mM) to yield a lipase activity of 900 USP units (USPU/mL).

### *In Vitro* Lipolysis

*In vitro* lipolysis was carried out as described previously with minor modifications (17). LBF was weighed directly into a thermostat-jacketed glass vessel (Metrohm, Switzerland) before digestion medium was added (final concentration of LBF was 2.5% (*w*/*v*)). The formulation was dispersed for 10 min in the digestion medium using a propeller stirrer (450 rpm). During the dispersion phase, the pH was manually adjusted to pH 6.5 ± 0.05. The digestion was initiated by addition of lipase. A pH-stat (Metrohm 907 Titrando) was used to maintain a pH of 6.5 through titration with 0.2 M (LC- and IV LBFs) or 0.6 M (MC-LBFs) NaOH. Samples were taken after 60 min of digestion and treated with 5 μL/mL lipase inhibitor (0.5 M 4-bromophenyl boronic acid in methanol) to inhibit further lipolysis.

Digestion was performed with pancreatic extract and immobilized lipase (Novozyme® 435) in order to (i) compare the extent of digestion and (ii) select the concentration of immobilized lipase for performing *in vitro* lipolysis assays. Type IIIB-MC and IIIB-LC formulations were selected as representatives of the MC- and LC-LBFs, respectively. Both formulations were digested according to the standardized protocol, using 900 USPU/mL of pancreatic extract, and with different concentrations of immobilized lipase (125, 250 or 750 PLU/mL). The extent of digestion was determined by plotting the free fatty acid (FFA) release against time. The presence of pancreatic extract resulted in a more extensive digestion than the presence of immobilized lipase. Immobilized lipase concentrations above 125 PLU/mL resulted in a limited increase in FFA liberation (Fig. S1). However, higher concentrations (>125 PLU/mL) impeded stirring and homogenous sampling. Therefore, a concentration of 125 PLU/mL was selected to digest all LBFs for 60 min.

### Cell Culture

Caco-2 cells, obtained from American Type Culture Collection (Manassas, Virginia), were cultivated as described previously in an atmosphere of 90% air and 10% CO_2_ (12). Briefly, Caco-2 cells (passage 95 to 105) were seeded on permeable polycarbonate filter supports (0.45 μm pore size, 12-mm diameter; Transwell Costar, Sigma-Aldrich) at a density of 44,000 cells/cm^2^ in Dulbecco’s modified Eagle’s medium supplemented with 10% fetal calf serum, 1% minimum essential medium nonessential amino acids, penicillin (100 U/mL), and streptomycin (100 μg/mL). Monolayers were used for experiments between day 21 and 26 after seeding.

### Compatibility Studies

Caco-2 cells were incubated with components present during *in vitro* lipolysis. The conditions are presented in Table III. The selected concentrations represent ‘worst-case’ scenarios; while the *in vivo* situation is dynamic, these experiments were performed under static conditions with a high concentration of the test component for a relatively long time (2 h). Digestion medium was used as a control in all studies.

**Table III:** Conditions Tested for Compatibility with Caco-2 Cells

Condition	
Buffer	
HBSS (pH 6.5)	Control
Digestion buffer	Simulated intestinal fluid containing bile salts and phospholipids
Excipients	
1.25% (w/v)	Highest concentration of a single excipient used during *in vitro* lipolysis (Table II)
0.625% (*w*/*v*)	Common concentration of single excipient used during *in vitro* lipolysis (Table II)
0.125% (w/v)	Common concentration of single excipient used during *in vitro* lipolysis (Table II)
LBF	
2.5% (w/v)	Common LBF concentration used during *in vitro* lipolysis (17), formulations describe in Table II
Enzyme	
Pancreatic enzyme (900 USPU/mL)	Common concentration of pancreatic enzyme added to *in vitro* lipolysis experiments (17)
Immobilized enzyme (125 PLU/mL)	Concentration of immobilized enzyme required for digestion of LBF
FFA	
Caprylic acid (87 mM)	Highest concentration of MC FFA released in previous digestions (17)
Oleic acid (37.5 mM)	Highest concentration of LC FFA released in previous digestions (17)
Digested LBF	
LBF digested with immobilized enzyme for 60 min	Common concentration of LBF and time span of *in vitro* lipolysis experiments digested with an enzyme concentration compatible with Caco-2 cells

#### TEER

TEER measurements were used to identify cell monolayers that were suitable for transport studies. Before and after all permeability experiments, cells were washed with pre-warmed (37°C) Hank’s balanced salt solution (HBSS; 7.4) and equilibrated with HBSS for 15 min. Subsequently, the confluence and integrity of the cell monolayers were assessed by measuring TEER. Only monolayers with initial TEER values greater than 250 Ω.cm^2^ were used for compatibility studies.

#### [^14^C]-mannitol Permeability

The hydrophilic paracellular marker mannitol was used as a model compound to investigate the effect of the test components on the integrity of the Caco-2 monolayers. All solutions were pre-warmed to 37°C. After, equilibrating cells with HBSS for 15 min, the buffer was removed and the filters with the cell monolayers were transferred to wells containing 1.2 mL of fresh, pre-warmed HBSS (pH 7.4).

Transport studies were initiated by adding 400 μL of digestion medium spiked with [^14^C] mannitol and components present during *in vitro* lipolysis (Table III) to the apical chamber. For components that were not compatible with the cells, the impact of mucin as a protective barrier was evaluated; mucin from porcine stomach type III (50 or 150 mg/mL) was dissolved in digestion medium and 100–200 μL was added to the monolayers. The monolayers were then incubated at 37°C for 10 min before initiating the transport experiment by adding the tests solutions containing [^14^C]-mannitol (final volume 400 μL in the apical chamber).

In all the transport experiments, 600 μL samples were removed from the basolateral chamber after 30, 60 and 120 min, and replaced with fresh HBSS. The samples were analyzed in a liquid scintillation counter (1900CA TriCarb; PerkinElmer Life Sciences). The apparent permeability coefficient (P_app_) was calculated according to the following equation:

$$ {P}_{app}=\frac{dQ}{dt}x\ \frac{1}{A\ x\ {C}_{donor}} $$

where Q is the [^14^C] mannitol appearing in the acceptor compartment as a function of time (t), A is the surface area of the Transwell membrane (1.12 cm^2^), and C_donor_ is the initial [^14^C] mannitol in the donor compartment. P_app_ values below 0.5 × 10^−6^ cm/s were defined as reflecting confluent monolayers, whereas values between 0.5 and 1.0 × 10^−6^ cm/s or >1.0 × 10^−6^ cm/s reflected intermediate and high incompatibility, respectively.

#### Progesterone Permeability

Progesterone was used to investigate the impact of mucin as a diffusion barrier to lipophilic compounds. Similarly to mannitol, transport studies with progesterone (C_donor_ 25 μM) were performed in the absence and presence of mucin. Samples were analyzed using a HPLC (Agilent Technologies 1290 Infinity) with a Zorbrax Eclipse XDB-C18 column (4.6 × 100 mm) (Agilent Technologies). The injection volume was 20 μL. The mobile phase consisted of acetonitrile:sodium acetate buffer (pH 5) at 85:15 (*v*/v) and was used at an isocratic flow rate of 1 mL/min. The retention time of progesterone was 1.98 min.

### Statistical Analysis

Data are presented as mean values with standard deviation (*n* = 3). Statistical analysis was performed using one-way ANOVA followed by a Dunnett’s test. *P*-values of less than 0.05 were considered statistically significant.

## Results

### Controls

The assessment of the digestion medium on the monolayer showed that it was compatible with the Caco-2 model as it showed sufficiently low P_app_ values for mannitol (<0.5 × 10^−6^ cm/s). In addition, P_app_ values were similar to values obtained during incubation with HBSS (pH 6.5; Fig. S2) during an incubation of 2 h. The application of a protective mucin layer, either in a low or high concentration, did not considerably affect the permeability of mannitol (a hydrophilic model compound) or progesterone (a lipophilic model compound), showing that mucin could be used as protective barrier without compromising permeation (Fig. S3).

### Excipients

**Fig. 1:**
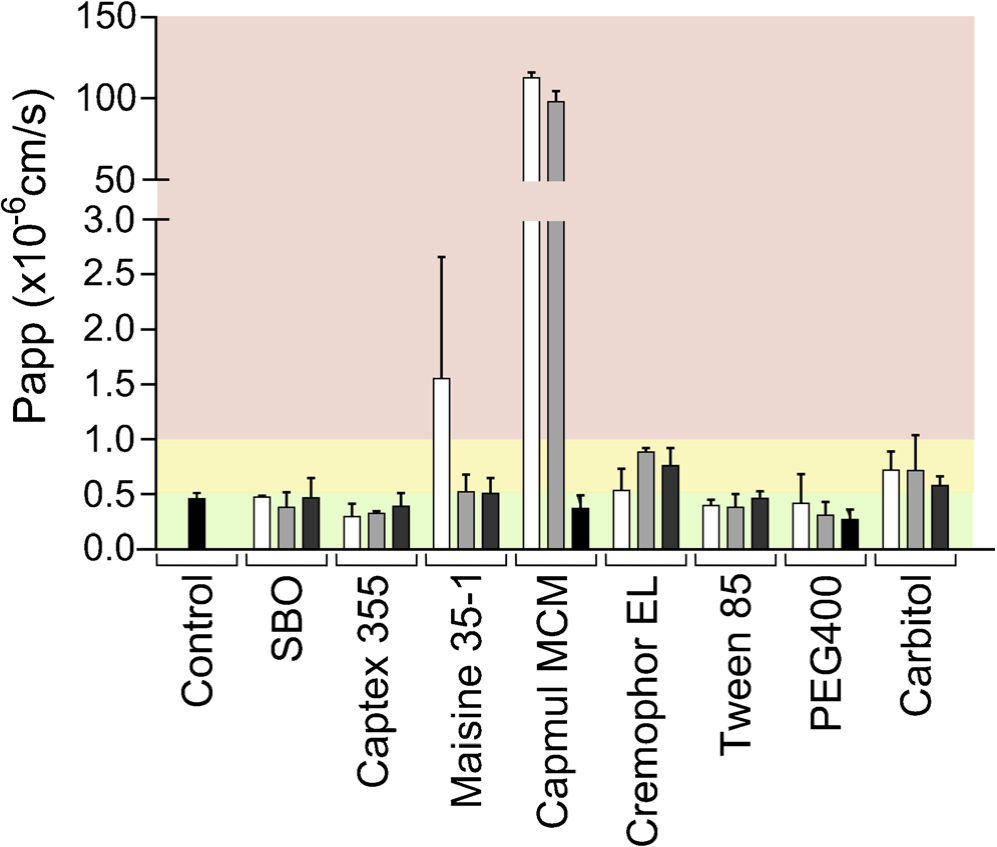
Effect of excipients on apical to basolateral transport of mannitol across Caco-2 monolayers. Bars represent average P_app_ values ± SD (*n* = 3). The black bar represent the control i.e. digestion medium. The white, light gray and, dark gray bars indicate excipient concentrations of 1.25, 0.625, and 0.125% (*w*/*v*), respectively. Red, yellow and green regions represent conditions that were not, intermediately and well tolerated.

Caco-2 cells were exposed to the single excipients used in the LBFs for 2 h (Fig. 1). Triglycerides were compatible with Caco-2 cells at all concentrations tested. The mixed glycerides showed a concentration-dependent incompatibility. Maisine 35–1 (mono-, di- and tri- LC glycerides) damaged the monolayer at a concentration of 1.25% (*w*/*v*) and Capmul MCM (mono-, di- and tri- MC glycerides) was incompatible already at low concentrations ≥ 0.625% (w/v). Cremophor EL and Carbitol showed intermediate tolerability at all concentrations whereas Tween 85 and PEG400 were compatible (Fig. 1).

### Lipid-Based Formulations

**Fig. 2:**
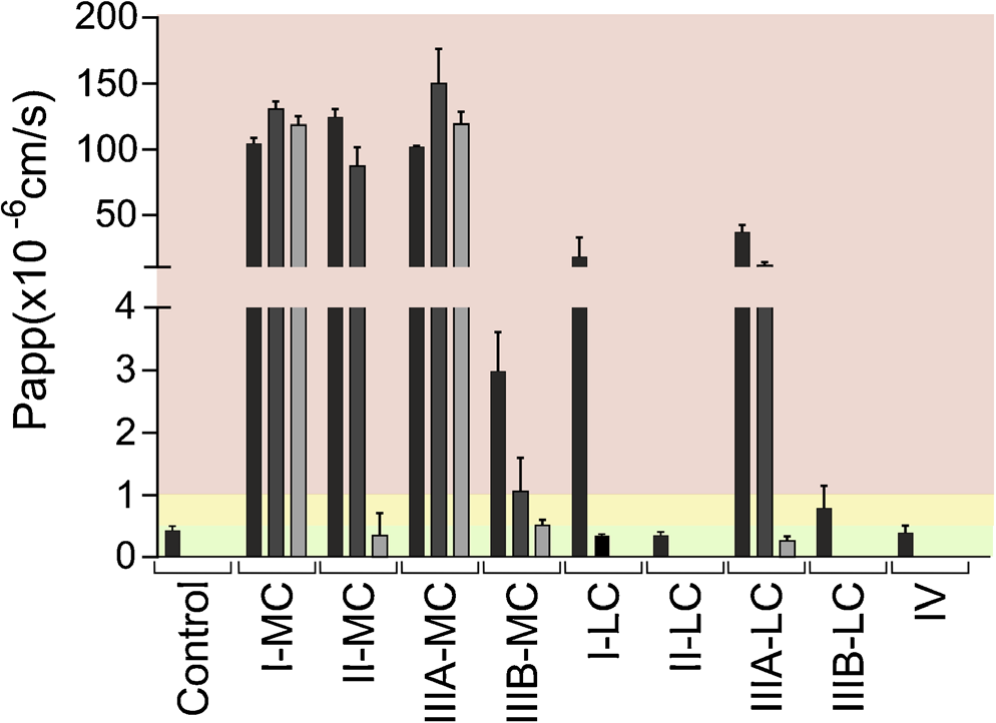
Effect of undigested LBFs on apical to basolateral transport of mannitol across Caco-2 monolayers. Bars represent average P_app_ values ± SD (n = 3). The black, dark gray, and light gray bars indicate the presence of no mucin, 100 μL of 50 mg/mL mucin, and 200 μL of 150 mg/mL mucin, respectively. Red, yellow and green regions represent conditions that were not, intermediately and well tolerated. The control was digestion medium.

Type II-LC and IV formulations were tolerated by the cells upon immediate exposure in relevant concentrations of 2.5% (w/v), i.e., no mucin was required to protect the monolayers (Fig. 2). For type IIIB-LC LBF P_app_ values indicated intermediate tolerability. The cell monolayer integrity was maintained by adding a low concentration of mucin (100 μL of 50 mg/mL) together with the type I-LC formulation whereas a higher concentration (200 μL of 150 mg/mL) was required to protect against the IIIA-LC. MC-LBFs were generally not compatible with Caco-2 monolayers (Fig. 2). However, the high mucin concentration (200 μL of 150 mg/mL) protected the monolayers against the damaging effects of the II-MC and IIIB-MC formulations.

### Enzymes

**Fig. 3:**
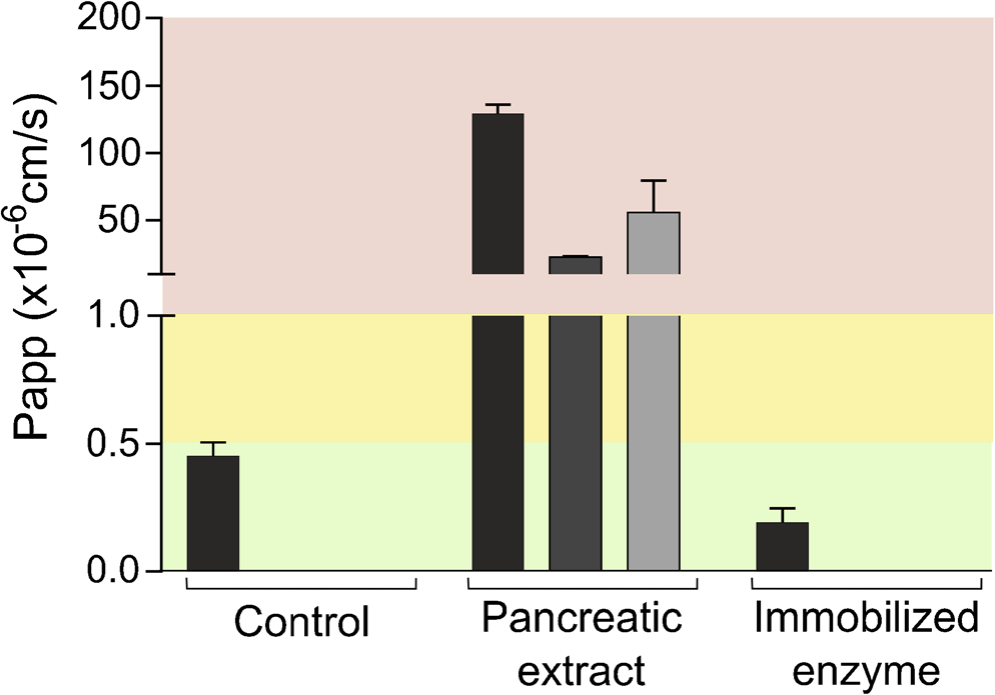
Effect of enzymes on apical to basolateral transport of mannitol across Caco-2 monolayers. Bars represent average P_app_ values ± SD (n = 3). The black, dark gray, and light gray bars indicate the presence of no mucin, 100 μL of 50 mg/mL mucin, and 200 μL of 150 mg/mL mucin, respectively. Red, yellow and green regions represent conditions that were not, intermediately and well tolerated. The control was digestion medium.

**Fig. 4:**
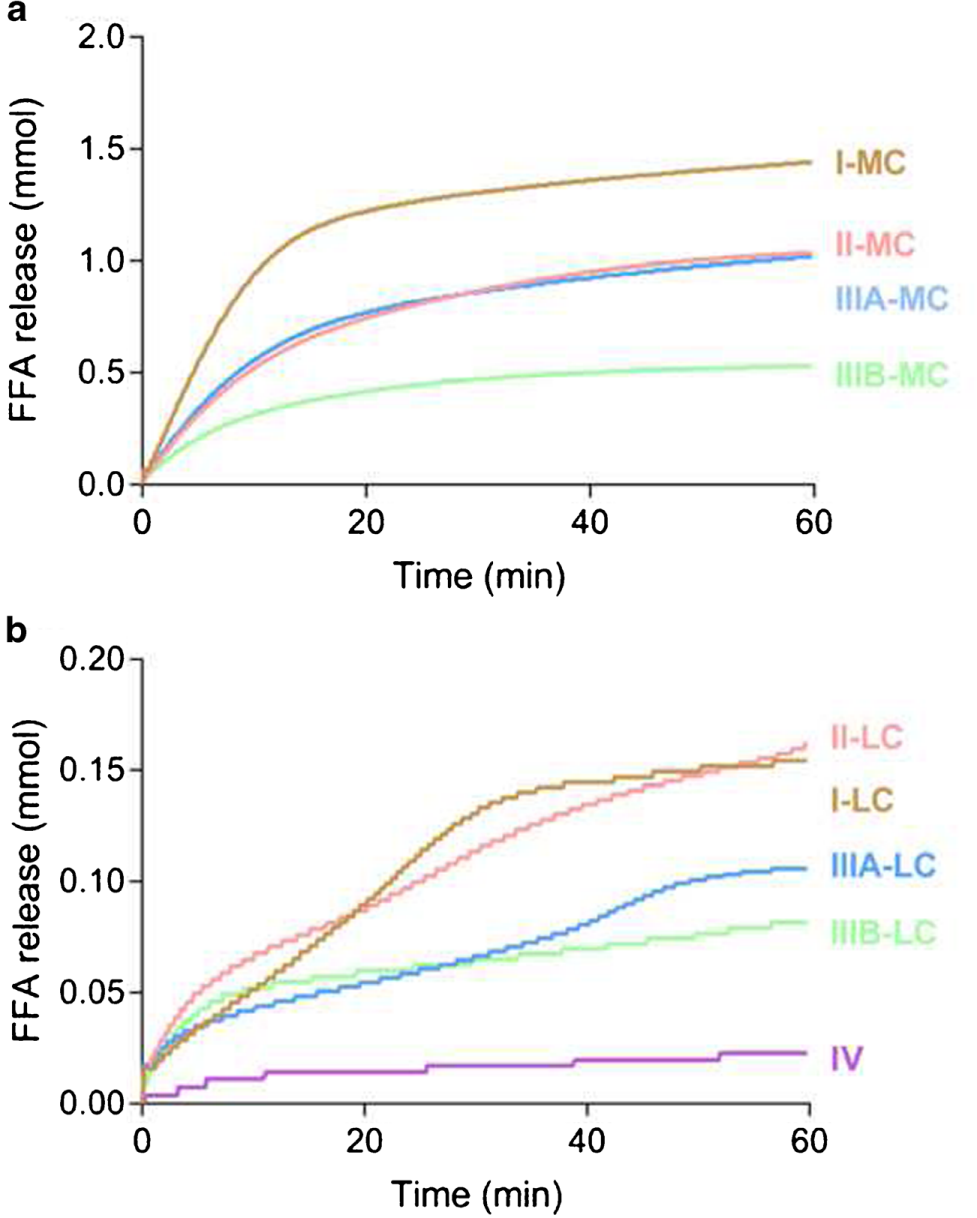
Apparent titration of FFA release during *in vitro* lipolysis (*n* = 1). (**a**) MC LBFs (**b**) LC-LBFs and IV-LBF.

The standardized *in vitro* method to assess lipolysis of LBFs, suggested by the LFCS consortium, uses an extract from porcine pancreas (17). However, this concentration of pancreatic extract (Table III) disrupted Caco-2 monolayers, even in the presence of mucin (Fig. 3). Therefore, the use of recombinant lipase immobilized on polymeric beads (Novozym® 435) was evaluated for the digestion of LBFs. A considerable release of FFA was observed during the digestion of all LBFs with 125 PLU/mL immobilized lipase (Fig. 4). Immediate exposure to this concentration of the enzyme was tolerated well by the cells (Fig. 3).

### Digestion Products

**Fig. 5:**
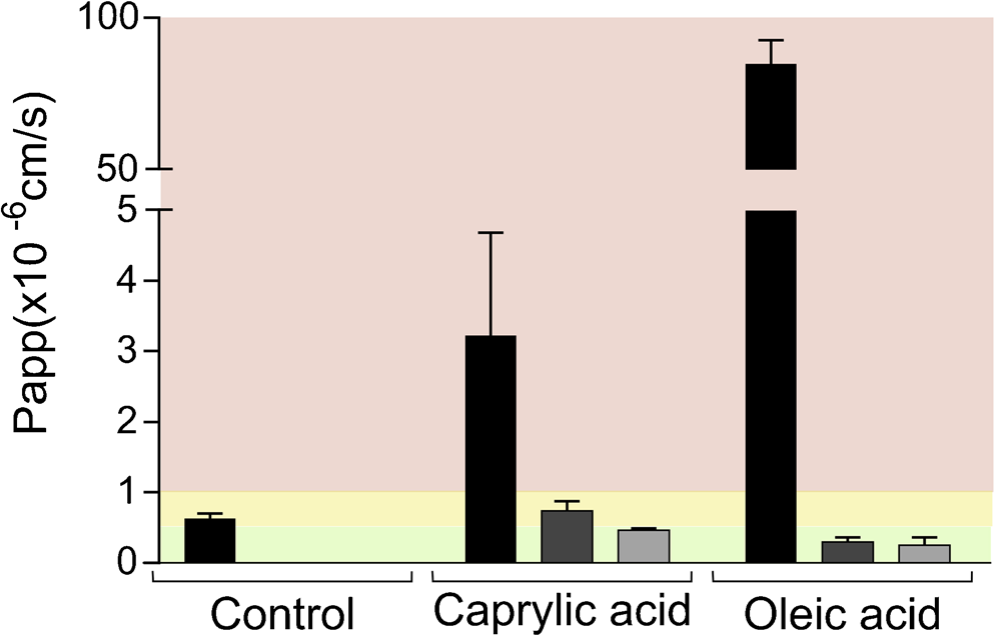
Effect of caprylic acid (87 mM) and oleic acid (37.5 mM) on apical to basolateral transport of mannitol across Caco-2 monolayers. Bars represent average P_app_ values ± SD (n = 3). The black, dark gray, and light gray bars indicate the presence of no mucin, 100 μL of 50 mg/mL mucin, and 200 μL of 150 mg/mL mucin, respectively. Red, yellow and green regions represent conditions that were not, intermediately and well tolerated. The control was digestion medium.

Caco-2 cells were exposed to caprylic and oleic acid representing digestion products released from MC- and LC-formulations, respectively. Mucin protected the cells against caprylic acid in a concentration-dependent manner. The highest concentration of mucin was required to shield the cells completely from its damaging effects. For the oleic acid, the lower concentration of mucin was already sufficient to protect monolayers against disruptive effects (Fig. 5).

### Digested Lipid-Based Formulations

**Fig. 6:**
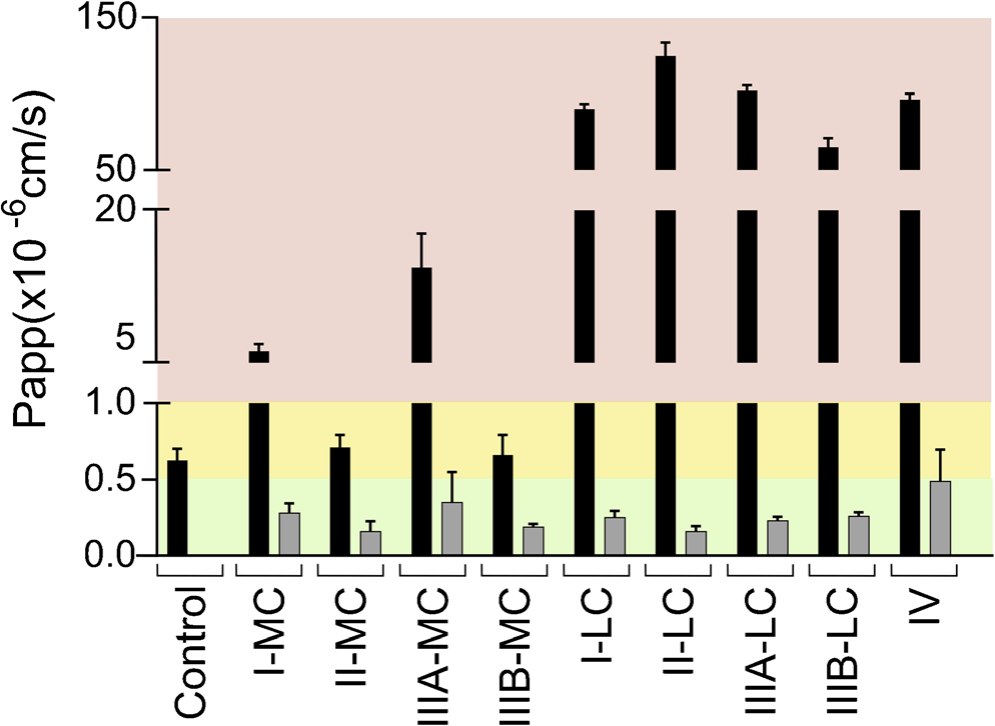
Effect of digested LBFs on apical to basolateral transport of mannitol across Caco-2 monolayers. Bars represent average P_app_ values ± SD (n = 3). The black and light gray bars indicate the presence of no mucin and 200 μL of 150 mg/mL mucin, respectively. Only the high mucin concentration was evaluated for protection of digested LBFs since the low concentration (100 μL of 50 mg/mL) has shown incomplete protection in previous experiments with single components of LBFs. Red, yellow and green regions represent conditions that were not, intermediately and well tolerated. The control was digestion medium.

LBFs were digested for 60 min to obtain media containing the excipients, immobilized lipase, and digestion products (Fig. 4). The MC formulations proved to be less damaging upon digestion than when administered in their undigested form (Figs. 2 and 6). Only intermediate monolayer damage was observed for the digested type II-MC and the IIIB-MC formulations. In contrast, all LC formulations and the type IV formulation exerted disruptive effects upon digestion whereas damaging effects were only observed for highly concentrated, undigested I-LC and IIIA-LC formulations (Figs. 2 and 6). Only the high mucin concentration (200 μL of 150 mg/mL) was evaluated for protection of digested LBFs since the low concentration (100 μL of 50 mg/mL) has shown incomplete protection in previous experiments with single components of LBFs. In the presence of high mucin concentrations, all digested formulations were compatible with the Caco-2 cells (Fig. 6).

## Discussion

Recent studies have identified the absence of an absorption compartment in the current *in vitro* lipolysis setup as a major reason for the poor prediction of the *in vivo* performance of LBFs (3,6,7). Therefore, the present study evaluated the compatibility between Caco-2 monolayers—the gold standard for intestinal absorption *in vitro* studies (11)—and components present during digestion studies. A number of excipients, LBFs, and digestion products, were shown to be tolerated by the cells at concentration levels relevant for evaluation of LBF performance. The pancreatic enzyme commonly used in standard lipolysis was found to be incompatible under all conditions tested, but immobilized lipase was endured by the Caco-2 monolayers at a concentration able to digest LBFs representing all classes of the LFCS (Figs. 3 and 4, respectively). Of particular interest is the fact that LBF concentrations used in digestion experiments could be applied without the need for dilution. Cells tolerated these concentrations during 2 h of incubation, which is significantly longer than a typical lipolysis experiment (30–60 min) (6,17). This opens up the possibility of coupling the lipolysis setup to an absorption chamber consisting of Caco-2 cells to perform lipolysis and absorption studies simultaneously.

P_app_ and TEER measurements have been used extensively to evaluate monolayer integrity of Caco-2 cells (11, 12, 19,20). These parameters correlated well in this study (i.e. a decrease in TEER corresponded to an increase in P_app_ of mannitol, Fig. S3 and S4). However, TEER measurements must be interpreted with caution. Variability in TEER can be introduced by fluctuations in temperature, medium formulation, passage number, and even the positioning of the electrodes. The most widely used system for measuring TEER consists of a pair of electrodes (known as chopsticks) and the electrodes only determine TEER locally (21). We therefore use the TEER measurements to identify cell monolayers that are suitable for transport studies, whereas we use a permeation marker such as mannitol or lucifer yellow to study monolayer integrity (12). When incubations resulted in mannitol P_app_ values below 0.5 × 10^−6^ cm/s, components were considered compatible with Caco-2 cells. However, some conditions resulted in P_app_ values that were below values obtained in the control condition (only digestion medium). A possible explanation is that some excipients including lipids can induce structural changes in the mucin, possibly resulting in decreased permeation (22).Vors *et al*. previously performed lipolysis experiments on emulsions followed by incubations on differentiated Caco-2 cells (13). However, to maintain the monolayer integrity they had to significantly dilute the digestion medium (1:20). Bu *et al*. exposed cells to 0.5% (*v*/v) LBFs and concluded that compatibility was influenced by the maturity of monolayers; differentiation was required for 21 days to optimize the survival rate (12,14). Both studies used LBF concentrations that were much lower than concentrations used in *in vitro* lipolysis experiments. Recently, Sadhukha *et al*. demonstrated the compatibility of Caco-2 cells with a selection of undigested LC formulations at concentrations relevant for *in vitro* lipolysis experiments. In agreement with the findings in the current study, they found that digested LC formulations showed significantly more incompatibility, i.e., lower TEER- and higher P_app_ values, than the corresponding undigested LBFs (15).

Cremophor EL and Tween 85 have previously been applied on differentiated Caco-2 cells as single excipients. In those experiments, a 2 h incubation of Cremophor EL (0.5% (v/v)) resulted in a slight decrease in cell viability according to a MTT test, but viability was still around 80% (14). Acceptable tolerance upon exposure to the Cremophor EL was also observed in our study, even after adding relatively high concentrations (0.625%–1.25% (*w*/*v*), Fig. 1). Up to 5% (w/v) Tween 85 was previously shown to be tolerated well by Caco-2 cells (23). This corroborates our observations as no damaging effects were observed at concentrations between 0.125 and 1.25% (w/v) (Fig. 1).

We demonstrated that the compatibility of LBFs and Caco-2 cells was highly influenced by formulation composition. A connection was observed between single excipient incompatibility and the effect of undigested LBFs on the cells. Undigested LBFs containing Capmul MCM, the excipient that caused severe integrity loss of the monolayers, were clearly more disruptive to the cells than other formulations (Table II and Fig. 2). Moreover, cells exposed to the IIIB-MC formulation, which contained the lowest fraction of Capmul MCM, performed better than the cells exposed to other MC-LBFs containing higher concentrations of this excipient. This is in agreement with previous data from Bu *et al*. who detected a decrease in toxicity when formulations contained higher amounts of Captex 355 in favor of Capmul MCM. In addition, they observed that formulations consisting of mixtures of mono-, di-, triglycerides and surfactants were better tolerated by monolayers than the single lipids or surfactants (14).

A clear connection between Caco-2 compatibility and the digestion process was detected. Overall, undigested MC formulations appeared to be more damaging to the cells than their digested counterparts (Figs. 2 and 6, respectively). Given that the critical micelle concentration (CMC) decreases with increasing chain length of the hydrophobic tails (25), the free concentrations of glycerides in dispersions of undigested MC-LBFs is relatively high. These free MC glycerides may insert into, and disrupt, membranes leading to lipid-induced rupture of the cells (14,25). Upon digestion, cells will be exposed to MC digestion products (MC FFA). Absorption enhancing effects of MC FFAs have been shown to occur in the vicinity of the CMC (19). Therefore, caprylic acid exhibited limited effects on membrane integrity when a much lower concentration (87 mM) than the CMC (225 mM) was applied (Fig. 6) (19,25).This may also provide an explanation for the mild membrane interactions observed for the digested MC-LBFs (Fig. 6), which when digested contained between 8.8 and 24.0 mM of ionizable FFA. On the contrary, components present in undigested dispersed LC-LBF will form micelles at relatively low concentrations resulting in limited exposure of glycerides to the Caco-2 monolayers. However, oleic acid (37.5 mM) as well as digested LC-LBFs severely damaged Caco-2 monolayers (Figs. 5 and 6, respectively). Upon digestion, released LC FFAs will be incorporated into the cell membrane and destabilize its lamellar phase through decreased transition temperature and the formation of inverted hexagonal phases (26). Oleic acid in particular, has been shown to affect membranes at mole fractions down to 0.025. Assuming (i) that the surface area occupied per phospholipid is about 64 Å^2^ (value for phosphatidylcholine) and (ii) the entire apical surface of the culture insert is covered by the monolayer (i.e. no paracellular transport route), only 0.005% of the LC FFA explored here would have to be inserted to exert a harmful effect (27,28).

Mucus, secreted by goblet cells, provides a protective barrier towards harmful endogenous and foreign substances *in vivo*. However, as Caco-2 cells originate from a colon cancer cell line, they do not always replicate the physiology of *in vivo* tissue and, for example, lack a mucus layer (29). Caco-2 cells could therefore be co-cultured with human mucus-producing cells to establish a mucus layer containing glycoproteins that mimic the protective barrier in the human gut (30). However, these co-cultures are relatively difficult to maintain and mucus layers can be easily removed during washing steps. Therefore, monolayers were in this study shielded against harmful effects by applying a protective porcine derived mucin layer on top of the cell barrier. This strategy has been used previously by Wuyts *et al*. who applied a barrier of mucin onto Caco-2 cells to protect them against fasted state human intestinal fluids (20). The concentration they used in their study was insufficient to completely protect the cells against some of the damaging effects, and so a larger amount of mucin was applied in this study (200 μL of 150 mg/mL mucin). Mucin has previously been found to significantly obstruct the absorption of lipophilic drugs in co-cultures of Caco-2 cells with mucin-producing HT29-MTX cells (31). Indeed, P_app_ of the lipophilic model compound progesterone was reduced in the presence of the low concentration of mucin but no statistical significant difference in P_app_ value was observed between the low and high mucin concentration. In both conditions, the transport was still considerable and the diffusion through the mucin layer was high (Fig. S5). P_app_ values obtained after the addition of mucin onto the Caco-2 cells while studying permeation of a selection of compounds (including lipophilic compounds) strongly correlated with P_app_ values obtained in the absence of mucin and with fractions absorbed in humans (20). Therefore, we do not expect the mucin to limit the usefulness of Caco-2 cells in combination with mucin in an absorption chamber for drug permeation studies.

Despite the protective barrier, Caco-2 cells were not able to tolerate the pancreatic extract. An alternative could therefore be to use artificial membranes to evaluate absorption of compounds during digestion of LBFs with the extract. For example, the biomimetic barrier Permeapad has been shown to maintain its permeation properties during the digestion of a type IIIA-LC LBF with the extract (32). However, we initially targeted the development of a cell-based model since Caco-2 cells enable both active and passive transport mechanisms to be explored (12). Although compounds formulated in LBFs typically cross the intestinal barrier through passive diffusion, other compounds, including bile salts and FFA, are substrates of transporters (33,34). Transporter-mediated uptake of these components changes the composition of the digestion medium and consequently its solvation capacity. It is therefore to be expected that cell-based systems more accurately capture the dynamics of the solubilizing intestinal lipoidal structures than e.g., artificial membranes.

In order to use Caco-2 cells during digestion studies, we found that the lipids need to be digested with immobilized lipase (Novozym® 435) instead of pancreatic extract. Novozym® 435 is a recombinant lipase B originating from *Candida Antarctica* that is immobilized on a macroporous polyacrylate resin. A modified *in vitro* digestion model employing this enzyme has been developed previously, showing that a similar extent of digestion could be obtained for the digestion of Captex 355 and Tricaprylin provided that the digestion lasts long enough. The activity of the immobilized lipase was shown to be independent of buffer or pH, enabling its use in protocols mimicking lipid digestion in different segments of the gastrointestinal tract (35). Other advantages are that immobilized lipase (i) enables easy separation from the digestion medium (ii) is reusable and, (iii) shows increased thermal stability. The activity of immobilized lipase is however slightly different to that of pancreatic extract (Fig. S1). This difference may be due to the specificity and affinity of the enzymes. The immobilized lipase consists of only one kind of lipase, whereas pancreatic extract contains a mixture of enzymes including phospholipase A2, colipase, and pancreatic lipase-related protein (35). In addition, access of the active site of immobilized lipase to triglycerides might be limited. Due to their low solubility in the aqueous phase triglycerides will mainly reside in the oil droplets and digestion needs to occur at the droplet interface. As immobilized lipase is confined to polymeric beads, it is likely that steric hindrance slows down digestion by this enzyme while pancreatic lipase that is dispersed freely in the digestion medium has easier access to the droplet interface. In contrast to human and porcine pancreatic lipase, the immobilized lipase does not display interfacial activation; i.e. a conformational change in the presence of a hydrophobic surface, resulting in exposure of the active site to the solvent. However, the active site is composed of the same catalytic triad consisting of serine, aspartic acid and histidine (36). As pancreatic extract in a concentration of 90 USPU/mL was shown to be compatible with Caco-2 cells in the presence of high mucin concentrations (Fig. S6A), the concomitant effects of 125 PLU/mL immobilized lipase and 90 USPU/mL of pancreatic extract on the digestion of IIIB-MC was evaluated (Fig. S6B). Unfortunately, combinations of the enzymes were incompatible with the cells (Fig. S6A) and had only limited effects on the extent of digestion (Fig. S6B).

Several studies have been undertaken to increase the physiological relevance of the *in vitro* lipolysis setup. For instance, as lipolysis is initiated in the stomach, a gastric lipolysis phase has been added (37). To capture the dynamics of the intestinal processes occurring after administration of a lipid-based drug delivery system an absorption sink needs to be added. As pancreatic extract is not compatible with Caco-2 cells (Fig. 3) we suggest to use immobilized lipase in the development of such a digestion model including absorption.

In the present study, pre- and post-digestion conditions tolerated by Caco-2 cells were identified in spite of the studies being designed as a ‘worst-case’ scenario with high concentration of natural and digested excipients being in contact with the cells for as long as 2 h. Hence, Caco-2 cell monolayers seem as a promising approach to study absorption simultaneously with lipolysis during performance testing of LBFs.

## Conclusion

We here demonstrated that Caco-2 monolayers are a promising tool in the development of a new method to couple *in vitro* lipolysis to an absorption compartment. The pancreatic extract typically used during lipolysis was damaging for the Caco-2 cell monolayers and so an immobilized lipase was used instead; it successfully digested the LBFs and was tolerated by the cell monolayers. Caco-2 cells, in combination with a protective mucin barrier, withstood all the undigested and digested LBFs explored herein except the undigested type I-MC and IIIA-MC formulations. These studies were performed in a ‘worst case’ scenario where the Caco-2 cells were exposed to high concentrations of all components for two hours. Digestion studies typically run for 30–60 min during which the condition is dynamic and we therefore expect that the Caco-2 cell model will perform even better under such circumstances.

## Electronic supplementary material

ESM 1 (PDF 691 kb)

## ABBREVIATIONS

FaSSIF: Fasted state simulated intestinal fluid
FFA: Free fatty acid
HBSS: Hank’s Balanced Salt Solution
LBF: Lipid-based formulation
LC: Long-chain
LFCS: Lipid formulation classification system
MC: Medium-chain
P_app_: Apparent permeability
TEER: Transepithelial electrical resistance
