# Supplementary material for: Caco-2 Cell Conditions Enabling Studies of Drug Absorption from Digestible Lipid-Based Formulations
Source: Pharm Res. 2018 Feb 26;35(4):74. doi: 10.1007/s11095-017-2327-8 (PMC5847224; doi:10.1007/s11095-017-2327-8)

Supporting information

Caco-2 cell conditions enabling studies of drug absorption from digestible lipid-based formulations

Janneke Keemink, Christel A.S. Bergström*

Department of Pharmacy, Uppsala University, Uppsala Biomedical Center, P.O. Box 580, SE-751 23 Uppsala, Sweden

*Corresponding author:

E-mail: christel.bergstrom@farmaci.uu.se

Phone: +46 18-471 4118

Department of Pharmacy, Uppsala University

BMC P.O. Box 580

SE-751 23 Uppsala

Sweden

Figure S1 Apparent FFA release during *in vitro* lipolysis of IIIB-MC (A) and IIIB-LC (B) with pancreatic extract (PE) or immobilized lipase (IE) in different concentrations.

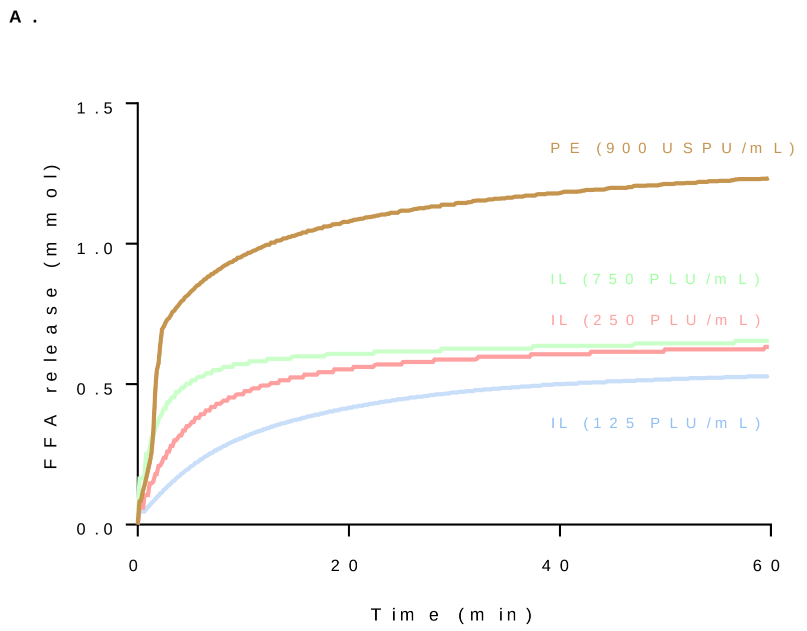

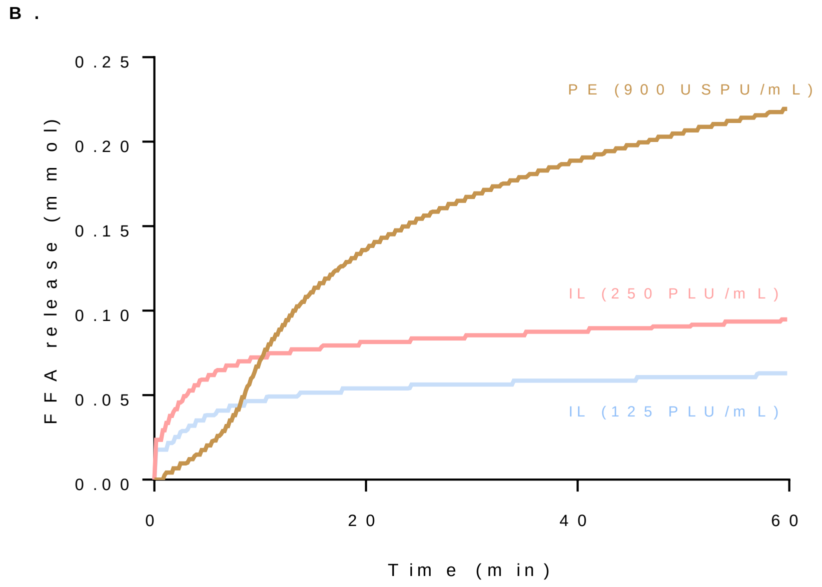

Figure S2 Effect of HBSS buffer (pH 6.5) and digestion medium on apical to basolateral transport of mannitol across Caco-2 monolayers. Bars represent average P_{app} values ± SD (n=3). Yellow and green regions represent conditions that were intermediately- and well-tolerated.

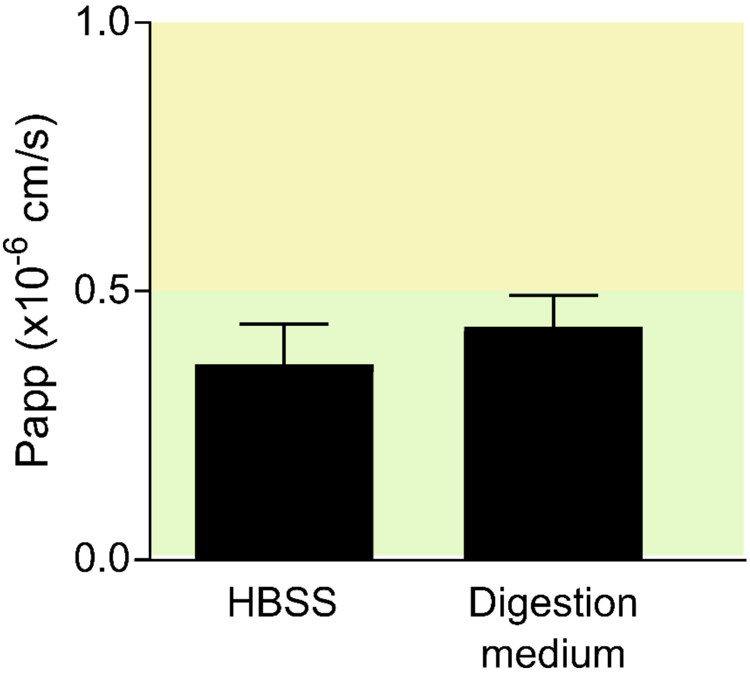

Figure S3 Effect of excipients on TEER values. Values represent average percentage of the initial TEER ± SD (n=3) after 2 h incubations. The black bar represent the control i.e. digestion medium. The white, light gray and, dark gray bars indicate excipient concentrations of 1.25, 0.625, and 0.125% (w/v), respectively. Components were considered compatible with the cells if TEER values were more than 75% of the initial value after incubation (green). Components resulting in a drop in TEER to values between 50 and 75% or below 50% of the initial value were considered to be intermediately (yellow) or poorly (red) tolerated.

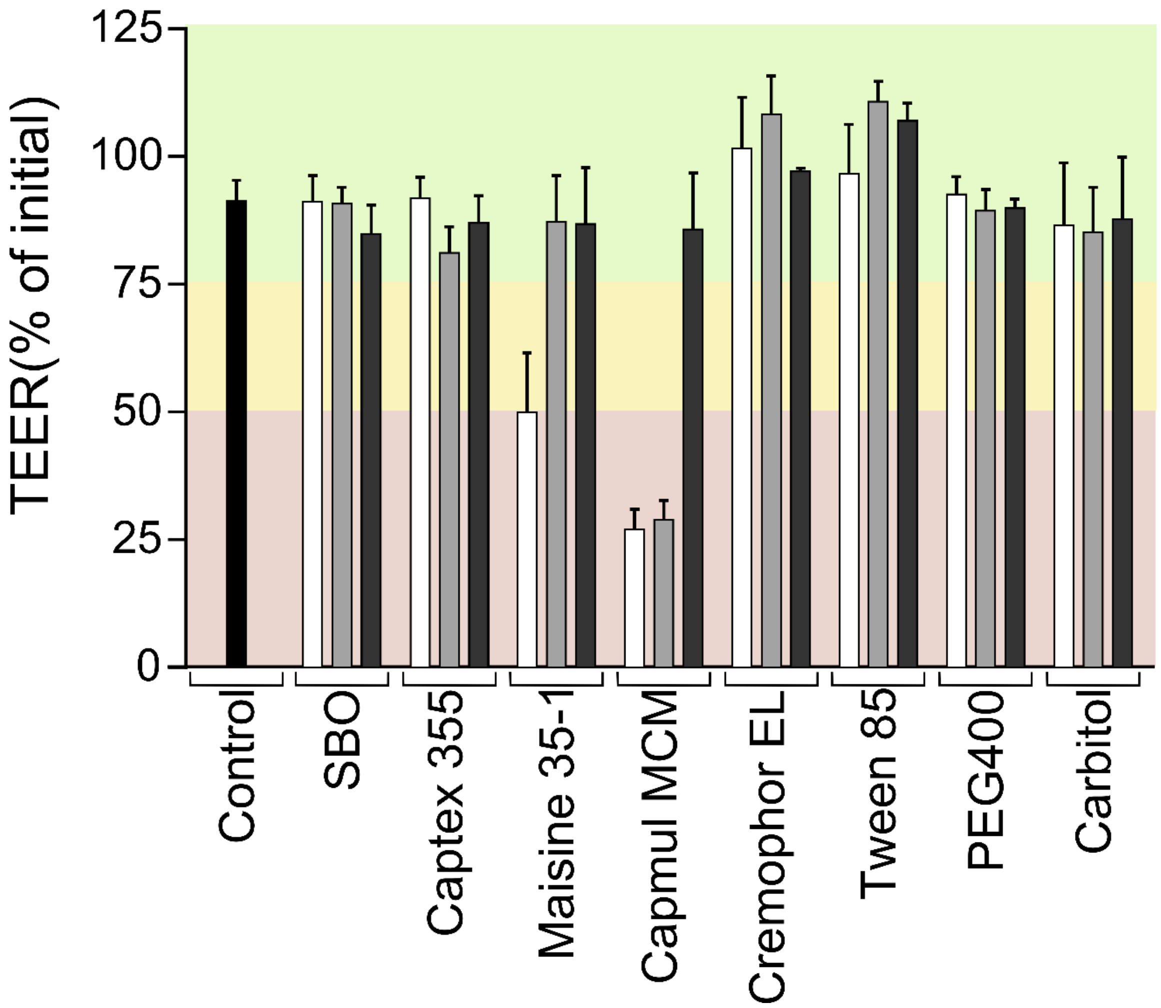

Figure S4 Effect of undigested LBFs (A), enzymes (B), free fatty acids (C), and digested LBFs (D) on TEER values. Values represent average percentage of the initial TEER ± SD (n=3) after 2 h incubations. The black, dark gray, and light gray bars indicate the presence of no mucin, 100 µL of 50 mg/mL mucin, and 200 µL of 150 mg/mL mucin, respectively. Components were considered compatible with the cells if TEER values were more than 75% of the initial value after incubation (green). Components resulting in a drop in TEER to values between 50 and 75% or below 50% of the initial value were considered to be intermediately (yellow) or poorly (red) tolerated.

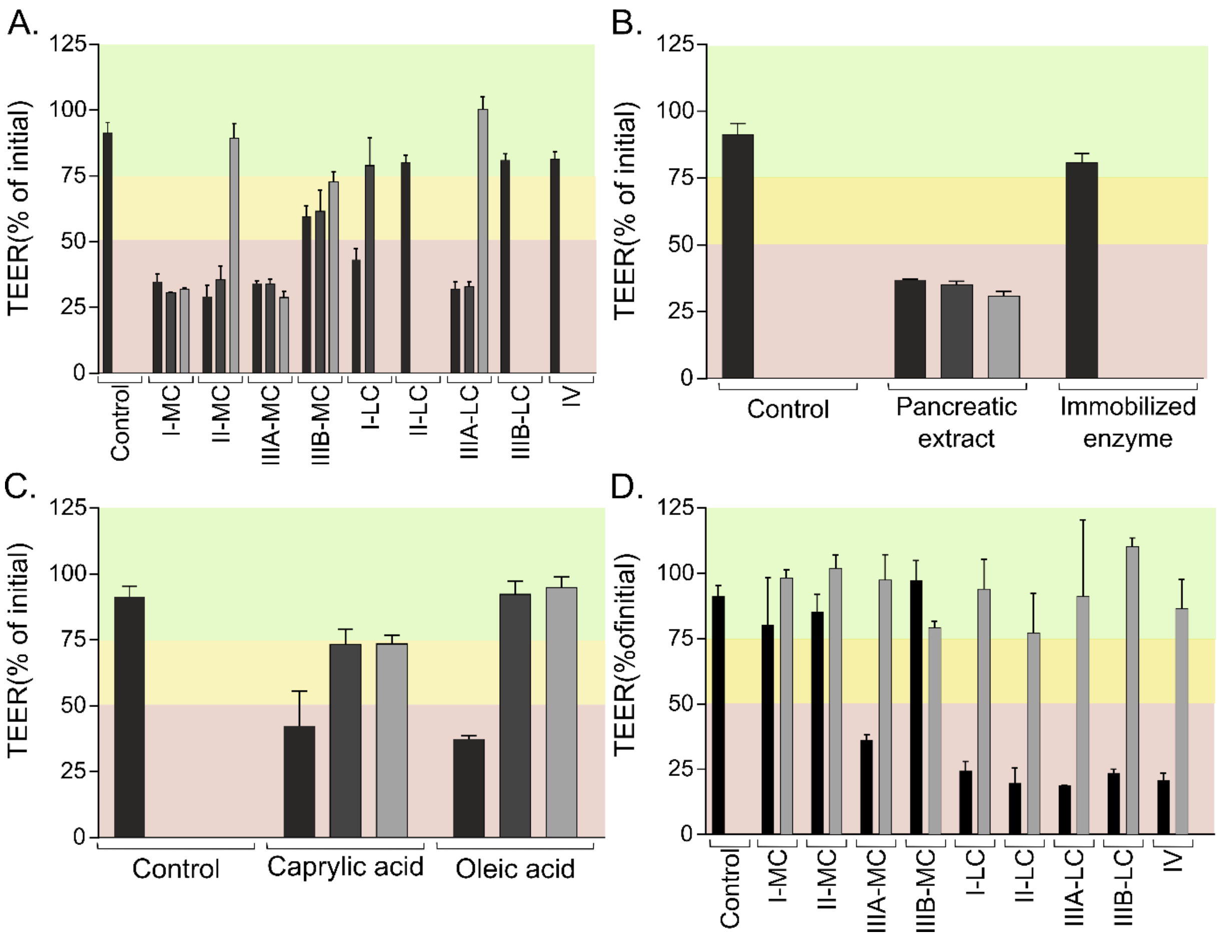

Figure S5 Apical to basolateral transport of progesterone across Caco-2 monolayers in the absence of mucin (control) and low (100 µL of 50 mg/mL) or high (200 µL of 150 mg/mL) mucin concentrations. Bars represent mean P_{app} values ± SD (n=3).

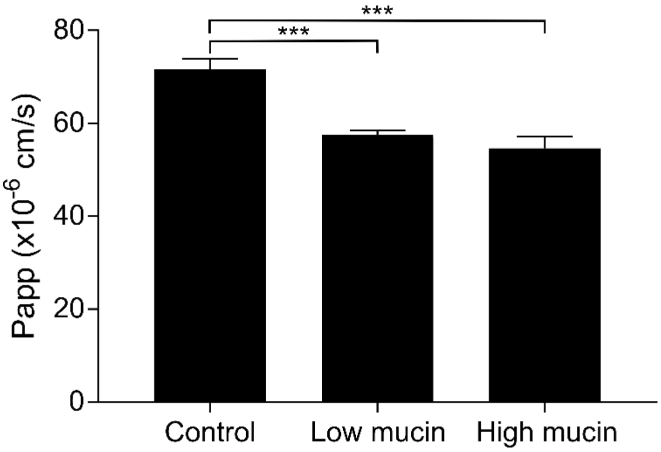

Figure S6 (A) Effect of different concentrations of pancreatic extract (PE) and a combination of PE and immobilized lipase (IL) on apical to basolateral transport of mannitol across Caco-2 monolayers in the presence of 200 µL of 150 mg/mL mucin. Bars represent average P_{app} values ± SD (n=3). Red, yellow and green regions represent conditions that were not, intermediately and well tolerated. The control was digestion medium. (B) Apparent FFA release during *in vitro* lipolysis of IIIB-MC with different enzyme concentrations.

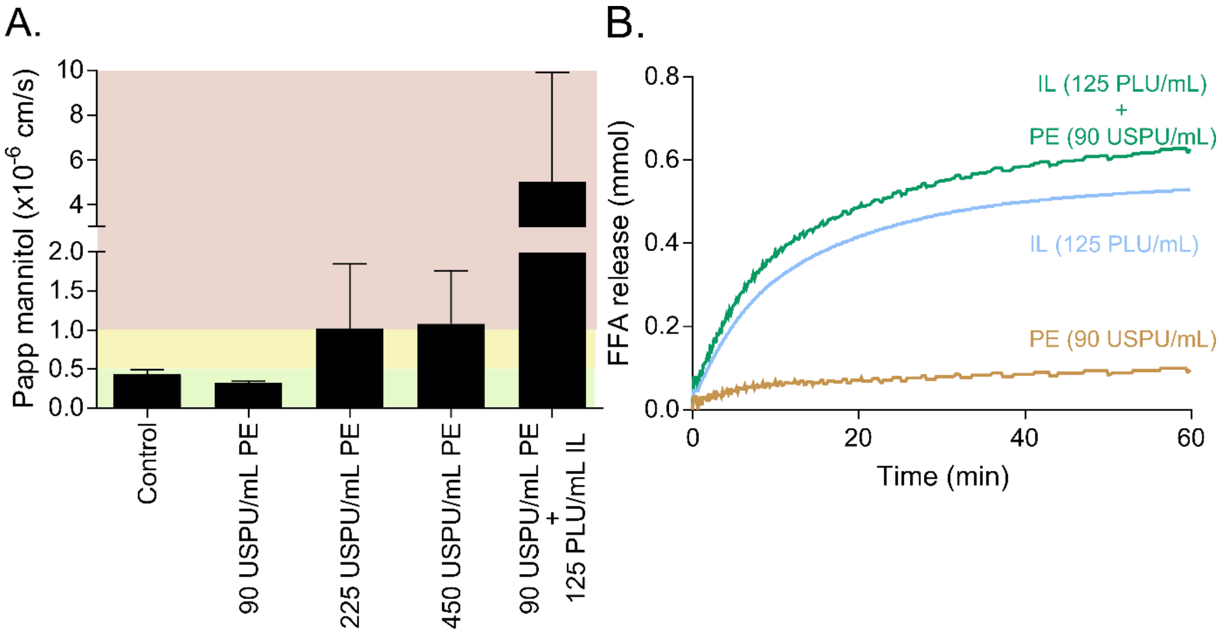

Supplement: Supplementary file 1 — (PDF 691 kb) [file 11095_2017_2327_MOESM1_ESM.pdf]
